# Do we need a reference standard for the muscle mass measurements?

**DOI:** 10.1002/ehf2.12356

**Published:** 2018-09-30

**Authors:** Nadja Scherbakov, Wolfram Doehner

**Affiliations:** ^1^ Center for Stroke Research Berlin CSB Charité ‐ Universitätsmedizin Berlin Berlin Germany; ^2^ Department of Cardiology (CVK), Deutsches Zentrum für Herz‐Kreislauf‐Forschung (DZHK) Charité ‐ Universitätsmedizin Berlin Berlin Germany; ^3^ Berlin‐Brandenburg Center for Regenerative Therapies (BCRT) Charité ‐ Universitätsmedizin Berlin Berlin Germany

Sarcopenia as a clinical term was suggested in 1988 by Irwin Rosenberg to refer an age‐dependent skeletal muscle wasting.^1^ In recent years, sarcopenia became more and more relevant in clinical practice. Beside the progressively aging population in our society, an increasing number of patients suffering from chronical diseases contributes to the growing prevalence of sarcopenia. Indeed, muscle wasting has been found in association with several diseases such as chronic heart failure,^2^ chronic kidney disease,^3^ chronic obstructive pulmonary disease (COPD),^4^ cancer,^5^ rheumatoid arthritis,^6^ diabetes mellitus,^7^ peripheral arterial disease^8^ etc. The consequences of decreasing muscle mass are wide‐ranging including metabolic dysregulation with insulin resistance and dyslipidemia, diminished bone mineral content, muscle structural changes with reduction of the neuromuscular junctions and muscle fibres switch, decrease of the fitness level up to frailty with increase in falls and functional disability.^9^ Muscle mass could be measured by several methods and mostly special technical equipment is required.

The recent publication “Pitfalls in the measurement of muscle mass: a need for a reference standard” by Buckinx *et al*. in *Journal of Cachexia, Sarcopenia and Muscle (JCSM)* investigated currently used methods for measurements of the lean body mass and muscle mass in order to determine a standard technique for use by clinicians and researchers.^10^ Therefore, members of the European Society for Clinical and Economic Aspects of Osteoporosis and Osteoarthritis working group performed a literature search between 2000 and 2016 on the role and methods of muscle mass measurements and the main recommendations were summarized in this publication. The methods of muscle mass assessments applied in multiple studies were compared for several key criteria such as safety, accuracy, feasibility, cost and availability. The main conclusion of this publication was to consider Dual X‐Ray Absorptiometry (DXA) as a ‘reference standard’ for assessment of the muscle mass. Other techniques including computed tomography, magnetic resonance imaging, bioelectrical impedance analysis (BIA), ultrasound, biomarkers, or anthropometric measures used for muscle mass assessments, all have a number of various considerations accounting for their limited applicability in clinical practice. Every technique has its advantages and limitations in different settings of clinical and scientific application and hence it is challenging to define a ‘gold standard’ for muscle mass measurements.

Yet, a question is to what extent are precise measurements of the muscle mass necessary to make a clinical diagnosis of sarcopenia? Indeed, a dominating role of the muscle function and muscle strength rather than muscle bulk in the diagnosing of sarcopenia has been proposed recently.^11^ Thus, several definitions of sarcopenia include two diagnostic criteria: (a) low muscle mass and (b) low muscle strength and/or muscle function.^12^, ^13^, ^14^, ^15^ Some of the consensus definitions even suggest starting diagnosing of sarcopenia with assessment of the muscle function or muscle strength and complete it by measurements of the muscle mass.^12^, ^13^ Thus, the Health, Aging and Body Composition (Health ABC) Study investigating 2,292 participants showed a high impact of quadriceps and handgrip strength on mortality while lean mass as assessed by DXA was not associated with mortality.^16^ The recent study by Locquet *et al*. comparing five screening methods for sarcopenia and investigating about 300 participants over two years, showed that the best results for identifying sarcopenic individuals were achieved if screening was performed with assessment of handgrip strength (a robust measure of muscle function), age and calf circumference (a surrogate of muscle bulk).^17^ Another study investigating 106 older patients with advanced cancer showed a positive association between muscle strength and overall survival at the beginning of chemotherapy.^18^ Clearly, muscle strength is the most relevant marker of muscle quality.^16^, ^19^ In contrast, muscle mass does not ultimately mean a good muscle function. Thus, a recent study investigating 140 adults over 65 years of age showed normal muscle volume and reduced handgrip strength in 13% of participants, mainly older females.^20^ In turn, in several interventional clinical trials increased muscle bulk was reported but this was not accompanied by significant increase in muscle functional capacity, rendering the respective therapeutic approach futile. Of course the role of muscle tissue as the body's main protein reservoir needs to be taken into account as appropriate and readily adaptive protein turnover is vital in multiple metabolic (anabolic capacity) and immune response processes (immune globulin synthesis). An age‐related reduction of muscle strength has been termed dynapenia.^21^ At tissue, cellular and molecular levels, sarcopenia‐related changes of skeletal muscle are similar to those in dynapenia: decline of the protein synthesis, increased oxidative stress, inflammation, alterations in the neuromuscular junctions or neurotransmitters, metabolic changes.^12^, ^21^, ^22^, ^23^, ^24^ However, only a combination of techniques applied for the muscle mass measurements may provide both information on the muscle mass and muscle quality.

Measurement of handgrip strength and gait speed are well established in clinical practice. However, a discrepancy of reference values is present between various definitions of sarcopenia. Thus, cutoff values for the handgrip strength range between less than 16 to 20 kg for women and between 26 to 30 kg for men.^12^, ^14^ This applies also to the gait speed with values less than 0.8 to 1.0 m/s and short physical performance battery (SPPB) with less than 8 to 9 points as a reference for the low muscle strength.^13^, ^15^, ^25^ Consequently, the reported prevalence of sarcopenia is wide‐ranging. Thus, a study investigating over 3,000 elderly women participating in the EPIDémiologie de l'OStéoporose study revealed a sarcopenia prevalence ranging between 3.3% and 20% depending on one of the six used definitions.^26^, ^27^ A recent meta‐analysis investigating over 58,000 individuals older than 60 years worldwide, reported a prevalence of sarcopenia ranging between 6% and 19% depending on sex, method of muscle mass assessment and geographic distribution.^28^ This meta‐analysis was based on three of the seven operational sarcopenia consensus definitions.^29^ Moreover, a prevalence of disease‐associated sarcopenia varies across clinical trials. For instance, in chronic heart failure a prevalence between 19.5% and 68%^2^, ^30^ and in COPD between 15% and 25%^31^, ^32^ has been described. Thus, a high prevalence of sarcopenia in clinical trials and registries is a common observation that requires better recognition as a relevant complication or comorbidity with consequent appreciation in comprehensive and holistic treatment concepts.

Nevertheless, sarcopenia is reversible. A recent observational study investigating 4,000 community‐dwelling older adults aged ≥65 years showed reversibility of sarcopenia in 20% and 14% of the patients at 2 and 4 years' follow‐up, respectively.^33^ Factors associated with the reversibility of sarcopenia were younger age, higher body mass index, absence of impairment in performing of instrumental activities of daily living.^34^ Surprisingly, neither physical activity nor protein level or vitamin D intake were associated with the reversibility of sarcopenia in this study.^34^ Another study, investigating 30 patients with gastrointestinal stromal tumour who were treated with imatinib, showed a reversibility of sarcopenia in 60% of the patients.^35^ A reversibility of disuse atrophy of type I and II muscle fibres 24 weeks after re‐use has been recently shown in an experimental study.^36^ In addition, resistance training is known to promote an improvement of muscle strength, muscle mass, quality of the muscle tissue, better physical performance and independence.^37^, ^38^ Therefore, recognizing sarcopenia by whatever method best suited to a specific clinical setting is highly relevant as it may impact on adequate treatment strategies and eventually lead to reduced frailty and better outcome.

## Conflict of interest

W.D. has received personal fees from Boehringer Ingelheim, Bristol‐Myers Squibb, Pfizer, Sphingotec, Vifor Pharma, and ZS Pharma as well as research support from Sanofi, Vifor Pharma and ZS Pharma. We acknowledge support from the German Research Foundation (DFG) and the Open Access Publication Fund of Charité – Universitätsmedizin Berlin.
